# Longitudinal study of Canadian correctional workers' wellbeing, organizations, roles and knowledge (CCWORK): Baseline demographics and prevalence of mental health disorders

**DOI:** 10.3389/fpsyt.2022.874997

**Published:** 2022-08-12

**Authors:** Bethany Easterbrook, Rosemary Ricciardelli, Brahm D. Sanger, Meghan M. Mitchell, Margaret C. McKinnon, R. Nicholas Carleton

**Affiliations:** ^1^Department of Psychology, Neuroscience and Behaviour, McMaster University, Hamilton, ON, Canada; ^2^Homewood Research Institute, Guelph, ON, Canada; ^3^MacDonald Franklin Operational Stress Injury Research Centre, London, ON, Canada; ^4^School of Maritime Studies, Fisheries and Marine Institute, Memorial University, St. John's, NL, Canada; ^5^Department of Criminal Justice, University of North Dakota, Grand Forks, ND, United States; ^6^St. Joseph's Healthcare Hamilton, Hamilton, ON, Canada; ^7^Department of Psychiatry and Behavioural Neurosciences, McMaster University, Hamilton, ON, Canada; ^8^Department of Psychology, University of Regina, Regina, SK, Canada

**Keywords:** mental health, wellbeing, correctional officers, occupational stress injuries, Correctional Services Canada

## Abstract

**Background:**

Researchers and practitioners have begun to recognize and empirically examine the mental health challenges facing public safety personnel (PSP). Empirical results from longitudinal data collection among PSP remains extremely scant, particularly for institutional correctional workers. We designed the current study to assess the mental health of Correctional Service of Canada (CSC) correctional officer recruits (CORs) across time to help clarify potential challenges to or protective factors for mental health across correctional officer (CO) careers.

**Methods:**

The current study uses data from the Canadian Correctional Workers' Wellbeing, Organizations, Roles, and Knowledge (CCWORK) study. The study uses a longitudinal design with self-report surveys administered online prior to CORs beginning the CSC Correctional Training Program. Initial baseline survey data were used to assess demographic information and mental health symptoms endorsed at the outset of the training program.

**Results:**

Participating CORs (*n* = 265; 40% female; age = 32.8, SD = 9.1) began training between August 2018 and July 2021. Participants were less likely to screen positive for one or more current mental health disorders (i.e., 4.9%) than previously published rates for serving correctional officers (i.e., 54.6%), including reporting lower rates of posttraumatic stress disorder (i.e., 2.4 vs. 29.1%) and major depressive disorder (i.e., 1.9 vs. 31.1%).

**Conclusion/Impact:**

Prevalence of positive screens for current mental health disorders in CORs appears lower than for the general population, and significantly lower than for serving correctional officers. The current results suggest an important causal relationship may exist between correctional work and detrimental mental health outcomes. Maintaining the mental health of correctional officers may require institutionally-supported proactive and responsive multimodal activities.

## Introduction

Correctional officers (COs) are frequently exposed to potentially psychologically traumatic events (PPTEs) in the workplace (1, 2). The occupational responsibilities of COs include violence prevention, riot control, response to fires, and pre-hospital emergency medical care (3, 4); as such, COs provide emergency services in federal penitentiaries that span the diverse roles of other public safety personnel (PSP; e.g., firefighters, public safety communicators, paramedics, police officers). COs are also responsible for the general care, control, and custody of incarcerated individuals (5, 6). The additional CO responsibilities are compounded by diverse additional chronic occupational stressors (7), including specific stressors like being tasked with staying in the same environment as, and providing care for, individuals involved in their PPTEs (3, 4, 8).

The CO exposures to PPTE and other occupational stressors appear to be associated with increased risk for clinically significant mental health challenges, including but not limited to symptoms associated with posttraumatic stress disorder (PTSD), major depressive disorder (MDD), generalized anxiety disorder (GAD), panic disorder, and substance use disorders (4, 8). Mental health challenges, including challenges consistent with one or more mental disorders, can be collectively described as posttraumatic stress injuries (PTSI) (9, 10). In a 2016 cross-sectional survey, most Canadian correctional workers (54.6%) screened positive for at least one mental health disorder, a rate higher than many other PSP groups (11). Correctional workers screened positive most often for either MDD (31.1%) or PTSD (29.1%) (11), and many reported high levels of past-year (11.0%) or lifetime (35.2%) suicidal ideation (12). There were similarly high rates reported in subsequent research with provincial correctional workers (13–15). Despite the prevalence of mental health disorders and suicidal behaviors among correctional workers, there remains a dearth of evidence regarding associated risk and potential protective factors among CORs who have yet to begin their careers in correctional services. Most of the available literature places a pathogenic focus on the health of correctional workers, uses cross-sectional designs that confound questions of causation, and limits progress to reduce adverse mental health outcomes among COs (16–20).

The Canadian Correctional Workers' Wellbeing, Organizations, Roles, and Knowledge (CCWORK) study is a longitudinal, multi-cohort research study with COs in Canada evaluating mental health, correctional work experiences, perceptions, aspirations, and training (21). CCWORK is the largest longitudinal study of Canadian correctional workers to date, using a rigorous longitudinal design to help address the numerous contemporary research gaps (21). The CCWORK objectives include: (1) understanding how self-reported CO mental health and mental health knowledge changes over time; and (2) identifying contextual factors that may influence CO mental health. CO mental health is evaluated over time from pre-training to post-training and then during the first ten years of participant careers. The CCWORK results will further enable researchers to identify contextual factors that may impact changes to CO mental health. Potential contextual factors identified through CCWORK include PPTEs inside and outside of the workplace, perceptions of safety within prisons, and challenges for mental health care (e.g., willingness to seek, availability). Understanding longitudinal changes in CO contextual factors and mental health outcomes may help clarify modifiable risk and protective factors. The CCWORK results may also inform our understanding of the complex interplay between contextual factors and correctional work for CO mental health.

In the current study, we examine baseline mental health characteristics of CO Recruits (CORs) collected during the Correctional Services Canada (CSC) Correctional Training Program (CTP), but prior to beginning their employment as a CO in the CSC. The results provide initial, baseline self-reported mental health information for CORs.

## Context

CSC employs over 18,000 individuals federally, with 7,800 employees working as correctional officers (22). Prior to beginning work as a CO, individuals must first pass through an in-depth recruitment process, that includes psychological assessments, followed by the CTP. The CTP has three required stages of completion: (1) an online training course; (2) online assignments; and 3) a 14-week intensive training program completed in-person (23). The CTP provides a breadth of theoretical and hands-on training for CORs. In 2018, the CTP was redesigned and added content and training focused on mental health and de-escalation. After completing the CTP, CORs are eligible for employment in federal correctional facilities as COs.

Once employed by CSC, a variety of operational demands are required of COs during their tenure. These demands may include: working lengthy shifts, some from 12 to 16 h, which may lead to inconsistency in scheduling, working within a hierarchical system, increasing the possibility of interpersonal conflict (i.e., similar structure to policing and armed forces), high stress interactions with incarcerated individuals, coworkers, superiors, and other individuals employed by CSC, administrative duties, and public scrutiny.

## Methods

### Participants and data collection

Data were obtained as a part of the currently ongoing prospective, longitudinal CCWORK Study (21). Data were collected between August 2018 and July 2021 using an internet-based, self-report survey administered through Qualtrics, and constitute baseline mental health prevalence screens for CORs beginning the CTP. The survey followed previously established guidelines for web-based surveys (24). CSC invited all recruits to participate in CCWORK via email correspondence during the early stages of CTP. CSC assisted with informing potential participants about the study, but COR participation was completely voluntary, and CSC employees never received access to any information participants shared with the research team. Participants were included if they responded to email correspondence that they were interested in participating, and were currently scheduled to undergo the third stage of CTP training. Individuals who had already completed CTP training and were working at CSC locations at the time of email contact were excluded. CORs who were willing to participate created a unique access code before providing informed consent and being redirected to the survey.

CCWORK recruitment sites have changed based on where CSC trains their recruits. In 2018, we recruited participants attending the newly opened CSC National Training Academy in Kingston, Ontario, which was the only training academy at the time. A second training academy has since been opened in Prince Edward Island, and following a COVID-19 related study pause, we began recruitment at all CSC National Training Academy satellite sites, located in each regional location of CSC (Ontario, Quebec, Atlantic, Pacific and Prairie).

### Measures

The pretest survey (i.e., data collected prior to CORs beginning the CTP) contained 164 closed- and open-ended used to assess diverse aspects of COR experiences. The items were derived largely from psychometrically valid tools, with additional items included based on expert consensus.

#### Demographic variables

Demographic variables included previous correctional work or PSP experiences, current employment status, province or territory of residence, year of birth, biological sex, gender identity, sexual orientation, education background, marital status, children, and languages spoken.

#### Posttraumatic stress disorder

PTSD symptoms were assessed using the PTSD Checklist 5 (PCL-5) (25). The PCL-5 is a commonly used self-report tool that assesses PTSD symptoms during the past month based on the Diagnostic and Statistical Manual of Mental Disorder-fifth edition (DSM-5) (25, 26). The PCL-5 has previously demonstrated high internal consistency and test-retest reliability in multiple populations (27). Participants are asked to rate their symptoms across 20 items using a 5-point scale ranging from 0 (not at all) to 4 (extremely). The PCL-5 can determine a provisional diagnosis of probable PTSD by summing all 20 items (range 0 to 80) and using a cut-point score (27). A total score >32 and endorsement of each PTSD cluster was considered a positive screen for PTSD (21) in the current study.

#### Major depressive disorder

MDD symptoms were assessed using the Patient Health Questionnaire 9-item (PHQ-9) (28). The PHQ-9 is a commonly used self-report tool that assesses MDD symptoms during the past 2 weeks based on the DSM-5 (26). Participants are asked to rate their symptoms across nine items using a 4-point scale ranging from 0 (not at all) to 3 (nearly every day) (28). The PHQ-9 has been shown to have high sensitivity and specificity (29). A total score >9 on the PHQ-9 was considered a positive screen for MDD (29) in the current study.

#### Panic disorder

Panic Disorder symptoms were assessed using the Panic Disorders Symptoms Severity Scale, Self-Report (PDSS-SR) (30, 31). The PDSS-SR is a commonly used self-report tool that assesses panic disorder symptoms during the past week based on the DSM-5 (26) and has been previously shown to be a reliable and valid measure of panic disorder severity (30). Participants are asked to rate their symptoms across seven items using a 5-point scale ranging from 0 (never) to 4 (all of the time) (30, 31). A total score >7 on the PDSS-SR was considered a positive screen for panic disorder (32) in the current study.

#### Generalized anxiety disorder

GAD symptoms were assessed using the General Anxiety Disorder 7-Item Scale (GAD-7) (33). The GAD-7 is a commonly used self-report tool that assesses GAD symptoms during the past week based on the DSM-5 (26). Participants are asked to rate their symptoms across seven items using a 5-point scale ranging from 0 (not at all) to 3 (nearly every day). The GAD-7 was has good reliability, as well as good criterion, construct, factorial, and procedural validity (33). A total score >9 on the GAD-7 was considered a positive screen for GAD (34) in the current study.

#### Alcohol use disorders identification test

Alcohol use and dependence were assessed using the Alcohol Use Disorders Identification Test (AUDIT) (35), a 10-item self-report tool with high validity, sensitivity, and specificity covering the frequency of alcohol consumption, drinking behavior, and alcohol-related problems (35). Each item is scored from 0 to 4, with a maximum total score of 40. A total score of 8 or more was considered a positive screen for hazardous alcohol use in the current study.

#### Cannabis use disorder identification test

Hazardous cannabis use and dependence were assessed using the Cannabis Use Disorder Identification Test-Revised (CUDIT-R) (36), an 8-item self-report tool with high sensitivity and specificity (36) covering frequency of consumption, abuse, dependence, and as well as psychological symptoms. For the current study the word marijuana was used in the scale rather than cannabis, in an effort to facilitate participants engagement. Items are scored from 0 to 4, with a maximum total score of 32. A total score of 12 or more was considered a positive screen for a cannabis use disorder in the current study.

#### Lifetime mental health disorders

Participants were also asked a series of open-ended and closed-ended questions regarding whether they had ever been diagnosed with several other mental health disorders including PTSD, MDD, panic disorder, GAD, persistent depressive disorder (PDD), bipolar I disorder, bipolar II disorder, cyclothymic disorder, social anxiety disorder, and obsessive-compulsive disorder. For additional details regarding study methods, please see Ricciardelli et al. (21).

### Statistical analysis

We conducted analyses using SPSS Version 26 software (IBM Corp, Armonk, NY, USA, 2020). All analyses were conducted using baseline data collected at the beginning of the longitudinal study. No follow-up data has been included in this manuscript. Baseline descriptive statistics are reported for each variable, along with overall estimates of positive screens for probable mental health disorders. We calculated means and standard deviations for each variable of interest, as well as medians and 95% confidence intervals where applicable. Participants were grouped into demographic categories including previous correctional work experience, previous PSP work experience, sex, gender, age, marital status, provincial region, and education for analytical comparisons.

## Results

Self-report baseline demographics of COR participants (*n* = 265) are presented in Table 1. The sample was approximately 60% men (*n* = 155), with a mean age of 32.8+/-9.1 years. Most participants (46%) were between 20 and 29 years of age, with 87% (*n* = 220) of this sample having completed at minimum some post-secondary education. In the full sample of respondents, 7.9% (*n* = 8) women/females and 3.2% (*n* = 5) men/males CORs screened positive for at least one current mental health disorder. Relatively few participants (7.9%; *n* = 21) reported a lifetime history of a mental health disorder diagnosis (Table 2). Approximately 2% of the sample screened positive for current symptoms of PTSD (*n* = 5), MDD (*n* = 4), or GAD (*n* = 4).

**Table 1:** Demographic information.

**Sociodemographic category**	**Any current positive screen % (*n*)**
**Gender (*****n*** = **256)**	
Men (*n* = 155)	3.2 (5)
Women (*n* = 101)	7.9 (8)
**Age (*****n*** = **257)**	
20-29 (*n* = 118)	5.9 (7)
30-39 (*n* = 82)	6.1 (5)
40-49 (*n* = 41)	0 (0)
50 and older (*n* = 16)	6.3 (1)
**Marital status (*****n*** = **252)**	
Married/common law (*n* = 125)	2.4 (3)
Single (*n* = 105)	7.6 (8)
Separated or divorced (*n* = 15)	13.3 (2)
Remarried (*n* = 5)	0 (0)
Other (*n* = 2)	0 (0)
**Provincial region (*****n*** = **253)**	
Ontario (*n* = 67)	1.5 (1)
Alberta (*n* = 59)	6.8 (4)
British Columbia (*n* = 31)	3.2 (1)
Manitoba (*n* = 27)	3.7 (1)
Quebec (*n* = 25)	16.0 (4)
New Brunswick (*n* = 16)	0 (0)
Nova Scotia (*n* = 14)	7.1 (1)
Saskatchewan (*n* = 14)	7.1 (1)
**Education (*****n*** = **252)**	
<4-year college/university (*n* = 98)	5.1 (5)
4-year college/university (*n* = 65)	6.2 (4)
Some Post-secondary (*n* = 53)	3.8 (2)
High school or equivalent (*n* = 32)	6.3 (2)
Completed graduate school (*n* = 4)	0 (0)

**Table 2:** Total sample current and lifetime mental health disorders.

**Mental disorder screening tools**	***n***	**Total sample; % (*n*)**
PTSD (PCL-5)	207	2.4 (5)
Major depressive disorder (PHQ-9)	212	1.9 (4)
Generalized anxiety disorder (GAD-7)	209	1.9 (4)
Panic disorder (PDSS-SR)	184	1.1 (2)
Alcohol use disorder (AUDIT)	175	0.6 (1)
Cannabis use	34	5.9 (2)
**Total number of current positive screens**	265	
0		95.1 (252)
1		3.4 (9)
2		1.1 (3)
3 or more		0.4 (1)
Any previous mood disorder diagnosis	211	4.3 (9)
Any previous anxiety disorder diagnosis	210	7.6 (16)
Any previous mental disorder diagnosis	265	7.9 (21)

*PTSD, posttraumatic stress disorder; PCL-5, posttraumatic stress disorder checklist for DSM-5; PHQ-9, patient health questionnaire-9; GAD-7, generalized anxiety disorder-7; PDSS-SR, panic disorder severity scale self-report; AUDIT, alcohol use disorders identification test*.

Many participants (33%; *n* = 87) reported previous work experience in correctional services, another PSP sector (e.g., border services, firefighters, paramedics, police, public safety communicators), with nursing, or with the armed forces. There were four participants with such previous work experiences (4.6%) who screened positive for a current mental health disorder (Table 3). There were 10 participants with such previous work experiences (11.5%) who reported having ever been diagnosed with any mental health disorder in their lifetime.

**Table 3:** Mental health disorders among participants with previous work experience in public safety, corrections, or armed forces.

**Mental disorder screening tools**	***n***	**Previous experience sample; % (*n*)**
PTSD (PCL-5)	75	2.7 (2)
Major depressive disorder (PHQ-9)	72	1.4 (1)
Generalized anxiety disorder (GAD-7)	72	2.8 (2)
Panic disorder (PDSS-SR)	64	1.6 (1)
Alcohol use disorder (AUDIT)	61	0 (0)
Cannabis use	10	10.0 (1)
**Total number of positive screens**	87	
0		95.4 (83)
1		2.3 (2)
2		1.1 (1)
3 or more		1.1 (1)
Any previous mood disorder diagnosis	72	4.2 (3)
Any previous anxiety disorder diagnosis	72	11.1 (8)
Any previous mental disorder diagnosis	87	11.5 (10)

*PTSD, posttraumatic stress disorder; PCL-5, posttraumatic stress disorder checklist for DSM-5; PHQ-9, patient health questionnaire-9; GAD-7, generalized anxiety disorder-7; PDSS-SR, panic disorder severity scale self-report; AUDIT, alcohol use disorders identification test*.

## Discussion

CCWORK is the first Canadian study to longitudinally assess the prevalence of mental health disorders among CORs, with the current study providing a cross sectional analysis of the mental health of CORs at occupational entry—a necessary baseline for the greater longitudinal study. The current results provide novel information regarding the baseline mental health of CORs prior to beginning work as COs in federal correctional facilities. The baseline results offer important insights into the mental health impact from CO service.

The prevalence of positive screens for current and previous lifetime mental health disorders (4.9 and 7.9%, respectively) among participating CORs appears lower than the diagnostic rates for the general Canadian population in 2019 (14%) (37) and lower than published results for actively serving COs (54.6%) (11, 13–15). The results provide initial evidence that CORs have a low prevalence of pre-existing mental health challenges when beginning their training, which suggests their subsequent work demonstrably negatively affects their mental health and that the psychological screening that is part of recruitment is effective. The results highlight the urgent need to identify risk and protective factors, and provide ready access to mental health services for all COs.

COs complete a standardized training process (with COVID adaptations as of 2020) across Canada; however, the profession remains very heterogeneous with diverse risk (e.g., types of PPTE exposures) and protective (e.g., available supports) factors that change during CO careers. CORs differ in age, marital status, and prior work experience, all of which may be relevant risk and possible protective factors for their mental health as COs. The type of mental health resources and support available to COs also differs substantially across Canadian provinces. The current CTP includes mental health training designed to increase awareness and support resilience, but there is no program evaluation evidence to support effectiveness. The CCWORK study data collection will support subsequent analyses assessing for associations between diverse demographic factors and mental health outcomes; however, there is a pervasive absence of effectiveness evaluations for mental health training and supports among PSP (38, 39), and the current results underscore the need for immediate longitudinal research.

Many COs understand that operational (e.g., workplace violence, PPTE exposures) and organizational stressors (e.g., shift work, administrative responsibilities) are inherent to their occupation, and many report experiencing or witnessing physical and verbal violence routinely in overcrowded, understaffed conditions (40). Correctional workers report elevated levels of job burnout compared to the general public and feeling overwhelmed by occupational demands, which can increase absenteeism and turnover, lower job satisfaction and involvement, and reduce workplace safety and security (41, 42). Operational and organizational stressors have each been associated with mental health challenges for correctional workers, and there may be important opportunities to support CO mental health by mitigating organizational stressors (7, 8). Pending results from the CCWORK study and future qualitative research into CO experiences may clarify how the diverse stressors interact with mental health after CORs become COs.

The current study has several limitations that can inform directions for future research. The low prevalence of positive screens in the current study precluded examining potential risk and resiliency factors at baseline; nevertheless, subsequent analyses with pending data from the CCWORK study yearly follow-ups may help to identify risk and resiliency factors. Rigorous pre-employment psychological health screening may have also accounted for the lower than anticipated prevalence rates of mental health disorders in CORs. The current study also focused on results from self-report screening tools rather than the pending clinical interview data, and as such, respondents may have underreported their mental health histories due to fear of negative career implications. The use of self-report may have also introduced unknowable response biases that affect the current results. Subsequent results based on clinical interview data and qualitative responses may provide additional insights into COR experiences that help to inform the current baseline and any changes to participant mental health that result from service. Future directions should also include the examination of functioning and work performance across the career span. The current study only included participating CORs from the federal correctional systems, limiting generalizability to provincial, territorial, or international conspecifics. Further, the SARS-CoV-2 pandemic occurred during the initial phases of data collection, which delayed recruitment, changed recruitment methods (e.g., telephone interviews rather than in-person interviews), and may have had an unknowable impact on participant mental health.

### Conclusions

The current results evidence Canadian CORs beginning the CTP as having fewer mental health challenges than the general public or COs with occupational tenure. Accordingly the current results in concert with results from the extant literature implicate CO work (i.e., operational and organizational stressors) as a probable causal factor associated with CO mental health challenges. The CCWORK study is designed to track mental health symptoms for participating COs during their careers, which should provide additional evidence of a causal relationship between service and mental health challenges, as well as helping to identify potentially modifiable risk and resiliency factors. Additional work examining workplace-specific risk factors for development of mental health challenges should support the development of a tailored, proactive and responsive programming to better support CO mental health.

## Data availability statement

The datasets presented in this article are not readily available because the data is confidential. Requests to access the datasets should be directed to rricciardell@mun.ca.

## Ethics statement

The studies involving human participants were reviewed and approved by Memorial University of Newfoundland. The patients/participants provided their written informed consent to participate in this study.

## Author contributions

BE and BS: formal analysis and writing—original draft and editing. RC: conceptualization, methodology, and writing—review and editing. MMc: conceptualization, methodology, clinical supervision, and writing—review and editing. RR: conceptualization, methodology, writing—review and editing, supervision, and funding acquisition. MM: conceptualization, writing—review and editing, table editing, and data collection. All authors agree to be accountable for the content of the work, and have critically revised the manuscript for submission.

## Funding

This study was supported by the Canadian Institutes of Health Research, Grant Nos. 411 385 (31 January 2019), 411 387 (31 January 2019), 422 567 (27 May 2019), and 440 140 (31 March 2020). The research was also supported by the Correctional Services of Canada and the Union of Canadian Correctional Officers (UCCO-SACC-CSN).

## Conflict of interest

The authors declare that the research was conducted in the absence of any commercial or financial relationships that could be construed as a potential conflict of interest.

## Publisher's note

All claims expressed in this article are solely those of the authors and do not necessarily represent those of their affiliated organizations, or those of the publisher, the editors and the reviewers. Any product that may be evaluated in this article, or claim that may be made by its manufacturer, is not guaranteed or endorsed by the publisher.
